# Effect of autologous platelet-rich plasma on the fertility and quality of cryopreserved buffalo bull semen: a comparative study using OptiXcell® and tris egg yolk extenders

**DOI:** 10.1186/s12917-024-04022-x

**Published:** 2024-06-07

**Authors:** Maha S. Salama, Mohey A. Ashour, Ehab S. Taher, Fatema Rashed, Ibrahim M. Ibrahim, Mohammad El-Nablaway, Ateya Megahed Ibrahim, Ostan Mihaela, Rada Olga, Nourelhuda A. Mohammed, Ahmed Abdeen, Mustafa Shukry

**Affiliations:** 1 Animal Reproduction Research Institute (ARRI), Agricultural Research Center (ARC), Giza, Egypt; 2 Animal Production Research Institute (APRI), Agricultural Research Center (ARC), Dokki, Egypt; 3 Riwina Animal Production Farm, Agricultural Research Center (ARC), Ministry of Agriculture, Kafrelsheikh, 33516 Egypt; 4 Department of Basic Medical and Dental Sciences, Faculty of Dentistry, Zarqa University, Zarqa, 13110 Jordan; 5 Sakha Animal Production Station, Agricultural Research Center (ARC), Ministry of Agriculture, Kafrelsheikh, 33516 Egypt; 6 Department of Basic Medical Sciences, College of Medicine, AlMaarefa University, 71666, Riyadh, 11597 Saudi Arabia; 7 Department of Administration and Nursing Education, College of Nursing, Prince Sattam bin Abdulaziz University, Al-Kharj, 11942 Saudi Arabia; 8 Family and Community Health Nursing Department, Faculty of Nursing, Port-Said University, Port-Said, 42526 Egypt; 9 Department of Biology, Faculty of Agriculture, University of Life Sciences King Mihai I, Timisoara, Romania; 10 Physiology and Biochemistry Department, Faculty of Medicine, Mutah University, Mutah, Al-Karak, 61710 Jordan; 11 Department of Forensic Medicine and Toxicology, Faculty of Veterinary Medicine, Benha University, Toukh, 13736 Egypt; 12 Department of Physiology, Faculty of Veterinary Medicine, Kafrelsheikh University, Kafrelsheikh, 33516 Egypt; 13 Department of Medical Biochemistry, Faculty of Medicine, Mansoura University, Mansoura, 35516 Egypt

**Keywords:** OptiXcell® extender, Tris egg yolk-based extender, Platelet-rich plasma, Buffalo bull semen, In vivo fertilization

## Abstract

**Background:**

Buffalo spermatozoa have a distinct membrane structure that makes them more vulnerable to cryopreservation, resulting in lower-quality post-thawed sperm. This decreases the success rate of artificial insemination in buffaloes. Understanding and addressing these specific vulnerabilities are essential for improving reproductive techniques in buffalo populations. The properties of cryopreserved buffalo bull semen were examined in this study regarding the impact of adding autologous platelet-rich plasma (PRP) to OptiXcell® or Tris egg yolk-based extenders. Ten buffalo bulls were used to collect semen. Each bull’s ejaculate was separated into two main equal amounts, each of which was then diluted with either OptiXcell® or Tris egg yolk-based extender, supplemented with various PRP concentrations (5%, 10%, and 15%), and the control (0%), before being cryopreserved according to established protocols. Following equilibration and thawing, the quality and functionality of the sperm were evaluated, along with the antioxidant enzyme activities (GSH and TAC), malondialdehyde (MDA) content, and in vivo fertilization rate of the thawed semen.

**Results:**

All PRP concentrations in both extenders, particularly 10% PRP, improved the quality and functionality of the sperm in both equilibrated and frozen-thawed semen. Additionally, the antioxidant enzyme activities in both extenders were higher in the PRP-supplemented groups compared to the control group in thawed semen (*P* < 0.05). All post-thaw sperm quality, antioxidant enzyme activities, and functionality aside from DNA integrity were higher (*P* *<* 0.05) in the PRP-supplemented OptiXcell® than in the PRP-supplemented Tris egg yolk-based extender. The fertility of cryopreserved semen in the extenders supplemented with 10% and 15% PRP increased (*P* < 0.05) significantly more than that of the control extenders, with 10% PRP being the optimum concentration in OptiXcell® (80%) compared to that of Tris egg yolk-based extender (66.67%) and control of two extenders (53.33% and 46.67%, respectively).

**Conclusions:**

Even though autologous PRP-supplemented extenders have a protective impact on equilibrated and cryopreserved semen, 10% PRP-supplemented OptiXcell® extenders are more effective at preserving post-thaw semen quality, functionality, and antioxidant capacity, which increases the in vivo fertility of buffalo bulls.

## Background

In contrast to other species, buffalo spermatozoa have a different membrane structure that renders them more susceptible to cryopreservation and produces lower-quality post-thawed sperm [1], consequently decreasing the success rate of artificial insemination (AI) [2]. Cryo-damage in the form of sperm membrane rupture, nuclear DNA and mitochondrial impairment, and producing reactive oxygen species (ROS) caused by the freezing and thawing processes causes a 50% reduction in sperm motility [3]. Therefore, the biochemical characteristics and quantity of cryoprotectants in semen extenders protect spermatozoa against cryoinjuries [4]. Supplementing semen extenders with autologous platelet-rich plasma (PRP) provides spermatozoa with the requirements they require to recover from cryopreservation damage; this could be due to the effects of PRP ingredients [5]. The platelet-rich plasma contains around 800 protein molecules, chemoattractants, hormones, and cytokines [6]. Recently, PRP has been effectively employed in reproductive biology, and it can be added to both fresh [7] and cryopreserved semen, including bucks [8], rams [9], buffalo bulls [10], and humans [11], with good consequences and enhancements in semen functionality and quality. Moreover, PRP has been injected into the testes of many species, including bucks [12], leading to improved sperm functionality and quality. In men, PRP treats infertility related to non-obstructive azoospermia [13]. It also enhances frozen-thawed sperm properties and testicular function in rabbits [14], improves sperm morphology and quantity in rats, renews rat testicular tissue, and maintains sex hormone balance [15]. PRP improves antioxidant protection, decreases oxidative stress (OS), and defends rat testicular tissues from damage [15]. PRP is rich in cytokines, including platelet-derived growth factor (PDGF), epidermal growth factor (EGF), fibroblast growth factor (FGF), transforming growth factors (TGF), insulin-like growth factor I (IGF I), nerve growth factor (NGF), vascular endothelial growth factor (VEGF), hepatocyte growth factor (HGF), and peptide hormones. PRP also contains ATP, calcium ions, zinc ions, histamine, serotonin, and superoxide dismutase (SOD). These components play a vital role in maintaining tissue and cell homeostasis. In the context of sperm quality and function, PRP’s factors have shown positive effects, such as enhancing sperm motility (TGF and VEGF), supporting sperm capacitation and acrosome reaction (zinc and calcium ions), protecting sperm from cryodamage, and improving post-cryopreservation quality (IGF-1, NGF, ATP, zinc ions, SOD, and platelet-activating factor) [11].

Ingredients of the extender seem to be one of the most crucial elements in the cryopreservation process because they preserve spermatozoa; similarly, egg yolk (20% v/v), which has been extensively employed as an impermeable cryoprotectant for the cryopreservation of spermatozoa in numerous species. Unfortunately, variations in egg yolk composition and the likelihood of microbial contamination affect sperm properties [16]. The preference is moving towards replacing the Tris egg yolk-based extender with one based on phospholipids or liposomes to lessen the harmful effects of egg yolk [17]. OptiXcell®, a commercial extender based on liposomes, has been shown to provide better cryoinjury protection than Tris egg yolk-based cryopreservation of bovine [18], equine [19], buffalo [20], and swine [21] semen.

As a result, we propose adding PRP to OptiXcell® and/or Tris egg yolk-based extenders to act as a cryodamage defense and improve the in vivo fertility of frozen-thawed buffalo bull semen. Furthermore, it is expected that the OptiXcell® extender will be more effective than the Tris egg yolk-based extender in the cryopreservation of buffalo bull semen. Consequently, this study aimed to compare the effects of OptiXcell® and Tris egg yolk-based extenders supplemented with various PRP concentrations on the quality and functionality of frozen-thawed sperm, as well as their antioxidant characteristics and in vivo fertility in buffalo-thawed spermatozoa.

## Materials and methods

### Ethical approval

The Institutional Animal Care and Use Committee of the Faculty of Veterinary Medicine at Kafrelsheikh University, Egypt allowed approval (IACUC#133/2021) for the present study involving the sampling.

### Reagents and chemicals

Unless otherwise stated, all compounds utilized in the current study were bought from Sigma Aldrich Co., USA.

### Experimental animals

The ethical committee of the Faculty of Veterinary Medicine, Kafrelsheikh University, Egypt, approved all the experimental techniques and procedures. Ten Egyptian buffalo (*Bubalus bubalis*) bulls (4–6 years old) were used; all were healthy, mature, fertile, and devoid of reproductive disorders and diseases. An artificial vagina obtained twenty-four ejaculates for every bull (two ejaculates per week). The animals (bulls and buffaloes) source as well as, the experiment was conducted at the Riwina Governmental Station, Animal Production Farm, Agricultural Research Center, Kafrelsheikh, Egypt, from September 2022 to November 2022.

### Platelet-rich plasma preparation and activation

To prepare the PRP, two centrifugations of blood were employed [22]. Briefly, PRP was obtained from the blood of the same ten buffalo bulls under sterile conditions on the same day as semen collection. Blood samples were collected in sterilized sodium citrate tubes (10 mL, Becton Drive, BD Vacutainer®, Franklin, USA) and then centrifuged for 5 min at 300 g during the initial centrifugation. The recovered plasma was recently re-centrifuged for 17 min at 700 g to separate the PRP. According to Gutiérrez et al. [23], a calcium gluconate (9.3 mg/mL) solution is required for PRP activation.

### Extender preparation

A Tris egg yolk-based extender was prepared using the following ingredients: 3.028 g Tris, 20 ml egg yolk, 1.678 g citric acid, 7.0 ml glycerol, 1.0 g glucose, 50,000 IU penicillin, and 25 mg gentamicin. These ingredients were liquefied in 73 mL of double-distilled water to make a final volume of 100 ml. The pH was adjusted to 6.8 [24] using a handheld pH meter (Twin pH, Horiba Ltd.) before adding the PRP. OptiXcell® was manufactured for use according to the manufacturer’s instructions and contains carbohydrates, antioxidants, buffers, phospholipids, glycerol, mineral salts, water, gentamicin, linomycin, specomycin, and tylosin (Ref. 026218, IMV Technologies).

### Semen cryopreservation

Ejaculates from bulls with a minimum of 85% normal sperm morphology and 70% or more motility are only used (170 ejaculates). Each bull’s ejaculate was separated into two identical portions, each diluted to 80 × 10^6^ (sperm/mL) using one of two semen extenders (OptiXcell® or Tris egg yolk-based). Each extender was added with several PRP concentrations (0, 5, 10, and 15%). The expanded semen samples were chilled (4 °C) for four hours for equilibration. After that, samples were put in 0.25 mL polyvinyl chloride straws (IMV, L’Aigle, France). The straws were placed in a biofreezer (Mini Digit-cool, ZH 400, IMV Technologies, L’Aigle, France) and frozen at -140 °C before being instantly submerged in liquid nitrogen for storage [25]. The frozen straws were thawed at 37 °C for 30 s for sperm evaluation.

### Equilibrated and post-thawed semen evaluation

#### Sperm motility, viability, and abnormalities

**Fig. 1:**
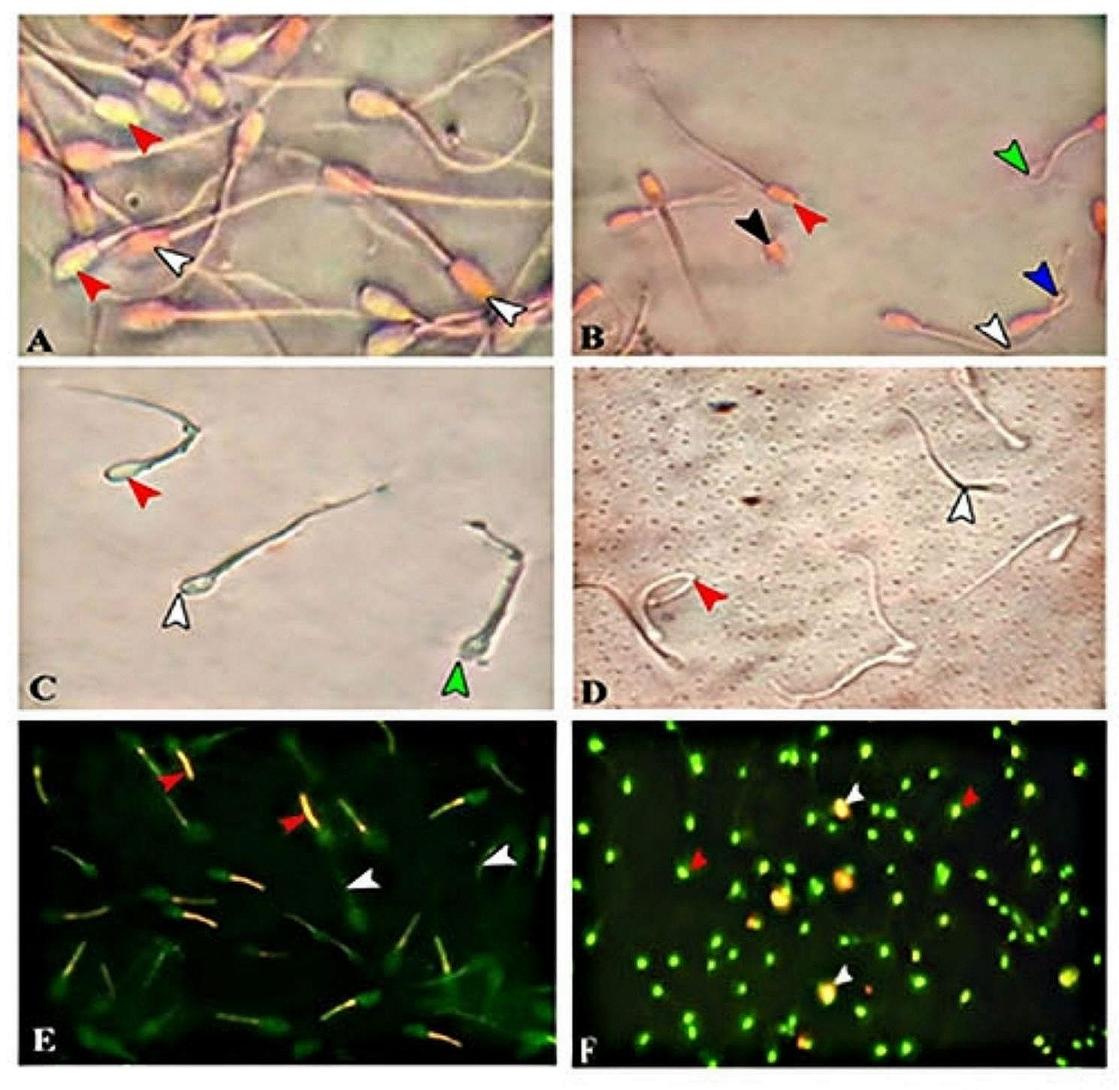
Images illustrating the quality and functionality of sperm. Figure 1. Panel **A**: Sperm viability: The red arrow indicates live sperm, and the white arrow indicates dead sperm. Panel **B**: Sperm abnormality: The red arrow indicates normal sperm, the blue arrow indicates a lobed tail, the green arrow indicates a coiled tail, the black arrow indicates a detached head, and the white arrow indicates a bent tail. Panel **C**: Acrosome integrity: The red arrow indicates an intact acrosome, the white arrow indicates a vacuolated acrosome, and the green arrow indicates a ruptured acrosome. Panel **D**: Membrane integrity: The red arrow indicates HOST-positive and the white arrow indicates HOST-negative. Panel **E**: Mitochondrial activity: The red arrow indicates high mitochondrial membrane potential (MMP), and the white arrow indicates low MMP. Panel **F**: DNA fragmentation: The red arrow indicates intact DNA, and the white arrow indicates a damaged DNA

Using a computer-aided sperm motion analyzer system (CASA; Sperm Vision TM software; Minitube) for progressive motility (%) investigations. Five µL of semen were put into a clean, previously warmed-up Makler chamber (37 °C). Eight microscopic areas were chosen at random and examined for each evaluation [1]. Sperm viability and abnormality percentages were assessed by mixing 5 µl of nigrosine (Sigma-Aldrich) and semen and applying it to a glass slide before drying above a Bunsen flame. An Olympus Bx-53 light microscope (Tokyo, Japan) was used to classify sperm cells as live or dead (400x). The heads of live sperm were clear, but those of dead sperm were red (Fig. 1, panel A) [26]. The same slide measuring sperm viability percentage was used to examine sperm abnormalities (Fig. 1, panel B) through an oil immersion lens. At least 200 spermatozoa were counted in five microscopic areas to determine sperm abnormalities and viability.

#### Acrosome integrity

To test the acrosome’s integrity (%), a formal citrate solution (2.92 g tri-sodium citrate dihydrate and 1 ml formaldehyde (37%) liquefied in 100 ml distilled water) was used [27]. In brief, 500 µL of the equilibrated or post-thawed semen specimen was combined with 50 µL of formal citrate solution. Five ml of the resulting mixture were put on a glass slide, coverslipped, and inspected under an oil immersion, 1000x bright-field microscope (Olympus Optical Co., Ltd., Japan). The proportion of acrosomal abnormalities (Fig. 1. Panel C; ruptured and vacuolated) in 200 spermatozoa was determined in 5 microscopic fields [28].

#### Membrane integrity

To evaluate the sperm plasma membrane’s functional integrity (%), the hypo-osmotic swelling test (HOST) was applied [29]. In detail, ten µL of semen suspended in 100 µL of 150 mOsm/kg hypo-osmotic solution (0.0735 g sodium citrate and 0.1351 g fructose dissolving in 10 mL Milli-Q water) were incubated in a water bath for 30 to 60 min at 37 °C. Post-incubation, sperm tail coiling or bending was evaluated by placing two µL of a thoroughly mixed sample onto a heated (38 °C) slide, covering it with an already-heated coverslip, and viewing the results using a phase-contrast microscope at 400 × magnifications. It was discovered that sperm cells with coiling and/or expanded tails had functioning plasma membranes (Fig. 1, panel D; HOST-positive).

#### Mitochondrial activity

The mitochondrial activity of sperm cells was measured using the lipophilic cation JC-10 (JC-10 Assay for Flow Cytometry, Sigma-Aldrich, St. Louis, MO, USA). JC-10 fluorescence reversibly transforms from green (a monomeric state) to orange (a multimeric state) as mitochondrial membrane potential improves. In summary, 1 × 10^6^ sperm/mL of equilibrated or thawed semen were placed into polypropylene tubes. The pellet was immersed in 500 µL of JC-10 and maintained at 37 °C for 1 h. After being washed with 1 mL of PBS and centrifuged for 10 min at 800 g, the samples were centrifuged again and resuspended in 1 mL of PBS. The samples were then subjected to flow cytometry analysis. Frequency plots were used to quantify the orange (JC-10 aggregates) or green (JC-10 monomers) fluorescence of the two populations using emission filters of 595 nm and 535 nm. The green color represented lower mitochondrial membrane potential (MMP), while the orange represented higher MMP (Fig. 1, panel E) [30].

#### DNA fragmentation

Evaluation of DNA integrity by the acridine orange assay, according to Martins et al. [31]. In brief, on glass slides, semen smears were prepared and then fixed in Carnoy’s solution (glacial acetic acid and methanol in a 1:3 proportion) overnight. The slides were air-dried and then incubated in the tampon solution (15 mmol/L Na2HPO4, pH 2.5, and 80 mmol/L citric acid) for 5 min at 75 °C. Afterward, the slides were stained with a 0.2 mg/mL acridine orange stain. After washing the slides with water, they were covered and assessed with an epifluorescence microscope. From each semen sample, 100 cells were examined. Fluoresced green indicated normal DNA content, but those with aberrant DNA fluoresced various colors, ranging from yellow-green to red (Fig. 1, panel F) [32].

### Biochemical parameter measurements of post-thawed semen

#### Glutathione reductase (GSH)

The GSH concentration of sperm was assessed using the technique developed by Beutler et al. [33]. Briefly, the semen samples were centrifuged at 1,000 g for 5 min at 22 °C after being precipitated with 50% trichloracetic acid (vol/vol). The reaction solution contained 0.25 mL of 5,5′-dithiobis-2-nitrobenzoic acid, 0.5 mL supernatant, and 2.0 mL of phosphate buffer. The solution was incubated at room temperature for 5 min. The GSH activity at 412 nm was measured with a spectrophotometer, and the levels of GSH were given in nmol/l [33].

#### Total antioxidant capacity (TAC)

According to Kumar et al. [34], the concentration of TAC was determined using a colorimetric assay kit (Catalogue No. K274, BioVision). This assay relied on Trolox as an antioxidant standard. The antioxidants converted Cu^2+^ ions to Cu^+^. The reduced Cu^+^ ions were then chelated with a colorimetric probe, resulting in a broad absorbance peak around 570 nm. This peak was proportional to the total antioxidant capacity and expressed as mmol/ml.

#### Malondialdehyde (MDA) concentrations

Malondialdehyde (MDA) concentrations in the semen samples were determined using the thiobarbituric acid reaction [35]. Briefly, a 500 µL aliquot of sperm was centrifuged for ten min at 800 g. The pellets were then collected, reconstituted in PBS, and re-centrifuged thrice. Finally, 1 mL of deionized water was added to the sperm, which was then snap-frozen and kept at -70 °C until analysis. The samples were thawed before the lipid peroxidation assay. Thiobarbituric acid-reactive substances were measured by comparing the absorption with the standard curve of MDA equivalents produced by the acid-catalyzed hydrolysis of 1, 1, 3, 3- tetramethoxypropane. MDA was represented as nmol/ml.

#### In vivo fertilization test

A total of 120 healthy cyclic (15 buffalo/PRP concentration/extender) buffalo were used, and each buffalo received two injections of 20 µg Busereline acetate (Receptal, GnRH agonist, Intervet International, Netherlands) at 9-day intervals, as well as a single injection of 750 pg cloprostenol sodium (Cloprostenol sodium, Estrumate, Coopers Animal Health L.T.D., Berkhamsted, England) on Day 7, and AI was performed on Day 10 for buffaloes [36]. 240 frozen-thawed straws (20 × 10^6^ spermatozoa) from two extenders were used for the insemination process. Each estrous buffalo received two inseminations, one in the morning and one in the evening. Insemination was done in December 2022, and on Day 45, after insemination, the linear probe ultrasonography was used to diagnose pregnancy in all inseminated buffalo.

### Statistical analysis

The data were statistically assessed via analysis of variance using SAS’s [37] General Linear Model Procedure. Differences between means were examined using Duncan’s multiple range test [38]. The Chi-square (χ^2^) test was used to investigate the pregnancy rate. All differences were applied at *P* < 0.05. OptiXcell® and Tris egg yolk-based extenders and conception rates were compared using a Student t-test.

## Results

### Effect of adding different concentrations of PRP-supplemented OptiXcell® and Tris egg yolk-based extenders on the quality and functionality of equilibrated semen

PRP-supplemented extenders positively affect sperm quality and functionality, especially when using 10% PRP-supplemented extenders during the equilibration period. There are no significant differences in semen quality and functionality between OptiXcell^®^ and Tris egg yolk-based extenders, except for MMP (%) of 5%, 10%, and 15% PRP-supplemented OptiXcell^®^ (Tables 1 and 2).

**Table 1:** Effect of PRP concentrations and extender types on equilibrated sperm quality (mean ± SEM)

Extender	PRP (%)	*N*	Progressive motility (%)	Viability (%)	Abnormality (%)
OptiXcell® ^A^	0%	48	57.17 ± 1.20^b^	79.67 ± 1.56^b^	13.83 ± 0.95^a^
	5%	48	60.33 ± 2.57^ab^	82.50 ± 1.28^ab^	11.83 ± 1.25^a^
	10%	48	64.33 ± 2.04^a^	85.50 ± 1.23^a^	7.50 ± 0.99^b^
	15%	48	62.83 ± 1.74^ab^	84.33 ± 1.33^a^	8.33 ± 0.96^b^
Tris egg yolk-based ^B^	0%	48	54.50 ± 0.76^c^	78.17 ± 1.60^b^	15.67 ± 1.33^a^
	5%	48	56.67 ± 0.80^bc^	79.83 ± 1.25^ab^	13.17 ± 0.98^ab^
	10%	48	61.50 ± 1.88^a^	82.83 ± 1.51^a^	10.83 ± 1.14^b^
	15%	48	60.17 ± 1.45^ab^	81.77 ± 0.98^ab^	10.33 ± 0.56^b^

*N* = 48 ejaculates. Within columns, values with different superscripts differ significantly for each parameter (*P* *<* 0.05)

Within the same column, means bearing a, b, and c differ among PRP concentrations (0%, 5%, 10% and 15%) within the same extender (OptiXcell® or Tris egg yolk-based). A and B differ between extender types (OptiXcell® or Tris egg yolk-based), at the same PRP concentrations (0%, 5%, 10% and 15%)

PRP, Platelet-rich plasma

**Table 2:** Effect of PRP concentrations and extender types on equilibrated sperm functionality (mean ± SEM)

Extender	PRP (%)	*N*	Acrosome integrity (%)	Plasma membrane integrity (%)	MMP (%)	DNA integrity (%)
OptiXcell®	0%	48	71.17 ± 1.85^b^	57.83 ± 1.30^b^	52.33 ± 2.43^b^	90.33 ± 1.59^b^
	5%	48	75.00 ± 1.57^ab^	61.17 ± 1.74^ab^	56.17 ± 1.35^abA^	93.33 ± 1.36^ab^
	10%	48	79.17 ± 2.12^a^	67.17 ± 2.18^a^	59.50 ± 2.43^aA^	95.00 ± 1.21^a^
	15%	48	77.50 ± 1.52^a^	65.5 ± 2.51^a^	58.33 ± 1.87^abA^	94.67 ± 1.09^a^
Tris egg yolk-based	0%	48	68.33 ± 1.38^b^	54.17 ± 1.76^c^	48.50 ± 1.59^b^	89.83 ± 1.42^b^
	5%	48	72.33 ± 1.52^a^	56.83 ± 1.45^bc^	50.17 ± 0.91^abB^	92.17 ± 0.60^ab^
	10%	48	76.17 ± 1.14^a^	62.17 ± 1.89^a^	53.00 ± 1.07^aB^	94.33 ± 0.62^a^
	15%	48	74.83 ± 1.17^a^	61.00 ± 1.51^ab^	51.83 ± 0.79^abB^	93.50 ± 0.43^a^

*N* = 48 ejaculates. Within columns, values with different superscripts differ significantly for each parameter (*P* *<* 0.05)

Within the same column, means bearing a, b, and c differ among PRP concentrations (0%, 5%, 10% and 15%) within the same extender (OptiXcell® or Tris egg yolk-based). A and B differ between extender types (OptiXcell® or Tris egg yolk-based), at the same PRP concentrations (0%, 5%, 10% and 15%)

PRP, Platelet-rich plasma; MMP, Mitochondrial membrane potential

### Post-thawed semen properties of control OptiXcell® and Tris egg yolk-based extenders

As shown in Tables 3, 4 and 5, and 6, except for DNA integrity (%), the control of OptiXcell^®^ extender has better sperm quality, functionality, antioxidant enzyme activity, and lower malondialdehyde levels (*P* *<* 0.05) than that of Tris egg yolk-based extender.

**Table 3:** Effect of PRP concentrations and extender types on post-thaw sperm quality (mean ± SEM)

Extender	PRP (%)	*N*	Progressive motility (%)	Viability (%)	Abnormality (%)
OptiXcell® ^A^	0%	48	46.50 ± 0.73^c^	75.50 ± 0.62^c^	18.67 ± 0.29^a^
	5%	48	49.17 ± 0.60^b^	78.67 ± 0.29^b^	15.17 ± 0.48^b^
	10%	48	52.34 ± 0.96^a^	80.83 ± 0.75^a^	9.67 ± 0.49^c^
	15%	48	51.17 ± 0.30^a^	80.17 ± 0.62^ab^	10.83 ± 0.38^c^
Tris egg yolk-based ^B^	0%	48	43.67 ± 0.53^c^	73.17 ± 0.40^b^	21.17 ± 0.48^a^
	5%	48	45.16 ± 0.58^bc^	74.50 ± 0.55^b^	18.93 ± 0.70^b^
	10%	48	47.25 ± 0.84^a^	76.67 ± 0.88^a^	14.17 ± 0.36^c^
	15%	48	45.67 ± 0.52^ab^	76.00 ± 0.41^a^	15.66 ± 0.22^c^

*N* = 48 ejaculates. Within columns, values with different superscripts differ significantly for each parameter (*P* *<* 0.05)

Within the same column, means bearing a, b, and c differ among PRP concentrations (0%, 5%, 10% and 15%) within the same extender (OptiXcell® or Tris egg yolk-based). A and B differ between extender types (OptiXcell® or Tris egg yolk-based), at the same PRP concentrations (0%, 5%, 10% and 15%). PRP, Platelet-rich plasma

**Table 4:** Effect of PRP concentrations and extender types on post-thaw sperm functionality (mean ± SEM)

Extender	PRP (%)	*N*	Acrosome integrity (%)	Plasma membrane integrity (%)	MMP (%)	DNA integrity (%)
OptiXcell®	0%	48	66.73 ± 0.36^cA^	46.50 ± 0.56^cA^	47.17 ± 0.31^dA^	87.73 ± 0.56^c^
	5%	48	70.67 ± 0.57^bA^	48.67 ± 0.42^bA^	51.83 ± 0.48^cA^	91.68 ± 0.28^b^
	10%	48	74.33 ± 0.49^aA^	52.30 ± 0.88^aA^	54.50 ± 0.23^aA^	93.50 ± 0.56^a^
	15%	48	72.83 ± 0.25^aA^	50.53 ± 0.33^bA^	53.17 ± 0.31^bA^	92.83 ± 0.21^a^
Tris egg yolk-based	0%	48	63.50 ± 0.76^cB^	43.82 ± 0.42^cB^	43.12 ± 0.70^cB^	86.67 ± 0.89^c^
	5%	48	66.44 ± 0.18^bB^	45.37 ± 0.72^bB^	46.59 ± 0.44^bB^	90.67 ± 0.94^b^
	10%	48	69.17 ± 0.70^aB^	47.87 ± 0.33^aB^	48.83 ± 0.60^aB^	92.67 ± 0.60^a^
	15%	48	67.77 ± 0.49^abB^	46.74 ± 0.62^abB^	47.33 ± 0.36^abB^	92.17 ± 0.37^a^

*N* = 48 ejaculates. Within columns, values with different superscripts differ significantly for each parameter (*P* *<* 0.05)

Within the same column, means bearing a, b, c, and d differ among PRP concentrations (0%, 5%, 10% and 15%) within the same extender (OptiXcell® or Tris egg yolk-based). A and B differ between extender types (OptiXcell® or Tris egg yolk-based), at the same PRP concentrations (0%, 5%, 10% and 15%). PRP, Platelet-rich plasma; MMP, Mitochondrial membrane potential

**Table 5:** Effect of PRP concentrations and extender types on post-thaw sperm antioxidant activity and malondialdehyde levels (mean ± SEM)

Extender	PRP (%)	*N*	GSH (nmol/l)	TAC (mmol/ml)	MDA (nmol/ml)
OptiXcell® ^A^	0%	48	0.71 ± 0.01^d^	19.67 ± 0.67^d^	23.91 ± 0.48^a^
	5%	48	0.95 ± 0.01^c^	21.33 ± 0.42^c^	18.23 ± 0.40^b^
	10%	48	1.49 ± 0.03^a^	25.17 ± 0.65^a^	11.77 ± 0.29^c^
	15%	48	1.42 ± 0.02^b^	23.33 ± 0.42^b^	12.75 ± 0.31^c^
Tris egg yolk-based ^B^	0%	48	0.63 ± 0.01^d^	17.61 ± 0.49^c^	26.28 ± 0.92^a^
	5%	48	0.75 ± 0.03^c^	19.77 ± 0.40^b^	23.78 ± 0.79^b^
	10%	48	1.31 ± 0.02^a^	22.83 ± 0.54^a^	17.50 ± 0.62^c^
	15%	48	1.16 ± 0.02^b^	20.60 ± 0.60^b^	19.18 ± 0.40^c^

*N* = 48 ejaculates. Within columns, values with different superscripts differ significantly for each parameter (*P* *<* 0.05)

Within the same column, means bearing a, b, c, and d differ among PRP concentrations (0%, 5%, 10% and 15%) within the same extender (OptiXcell® or Tris egg yolk-based). A and B differ between extender types (OptiXcell® or Tris egg yolk-based), at the same PRP concentrations (0%, 5%, 10% and 15%). PRP, Platelet-rich plasma; GSH, Glutathione reductase enzyme; TAC, Total antioxidant capacity; MDA, Malondialdehyde

**Table 6:** Effect of PRP concentrations and extender types on the conception rate of buffalo

Extender	PRP (%)	Conception rate (%)
OptiXcell®	0%	8/15 (53.33%)^c^
	5%	8/15 (53.33%)^c^
	10%	12/15 (80%)^aA^
	15%	10/15 (66.67%)^b^
Tris egg yolk-based	0%	7/15 (46.67%)^b^
	5%	7/15 (46.67%)^b^
	10%	10/15 (66.67%)^aB^
	15%	9/15 (60%)^a^

Number of inseminated buffalo = 15 buffalo per extender per PRP (%). Within a column, values with different superscripts differ significantly for each parameter (*P* *<* 0.05). Within a column, means bearing a, b, and c differ among PRP concentrations (0%, 5%, 10%, and 15%) within the same extender (OptiXcell® or Tris egg yolk-based). A and B differ between extender types (OptiXcell® or Tris egg yolk-based) at the same PRP concentrations (0%, 5%, 10% and 15%)

PRP, Platelet-rich plasma

### Post-thawed sperm quality

All concentrations of PRP-supplemented OptiXcell® extenders, as well as the 10% and 15% of PRP-supplemented Tris egg yolk-based extenders, significantly improved (*P* *<* 0.05) sperm motility, vitality, and reduction of sperm abnormality when compared to their control groups. PRP-treated semen at all concentrations supplemented with OptiXcell® demonstrated significantly (*P* *<* 0.05) better motility, vitality, and reduced abnormality compared to Tris egg yolk-based extenders (Table 3).

### Sperm functionality

In comparison to the control groups, semen extended with OptiXcell® and Tris egg yolk-based extenders supplemented with various PRP (5, 10, and 15%) concentrations showed significantly (*P* *<* 0.05) improved acrosome integrity (%), sperm plasma membrane integrity (%), DNA integrity (%), and mitochondrial membrane potential (%), as shown in Table 4. Except for DNA integrity (%), all concentrations of PRP-supplemented OptiXcell® significantly (*P* *<* 0.05) improved sperm functionality compared to the Tris egg yolk-based (Table 4).

### Antioxidant enzyme activity and malondialdehyde level

The antioxidant capacity was significantly (*P* *<* 0.05) increased, and MDA levels were lowered in PRP-supplemented OptiXcell® as compared to PRP-supplemented Tris egg yolk-based extender. Supplementation of OptiXcell® and Tris egg yolk-based extenders with various concentrations of PRP (5, 10, and 15%) significantly (*P* *<* 0.05) increased TAC and GSH and lowered MDA levels compared to controls (Table 5).

### In vivo fertilization rate (%)

The conception rate (%) was significantly (*P* < 0.05) higher in buffaloes inseminated with semen from 10% PRP-supplemented OptiXcell® than that from PRP-supplemented Tris egg yolk-based extenders. The conception rate (%) was significantly (*P* < 0.05) improved in 10% and 15% PRP-supplemented OptiXcell® and Tris egg yolk-based extenders compared to 5% PRP and control groups of the two extenders, with 10% PRP being the optimal concentration with cryopreserved semen in the OptiXcell® extender (80%) compared to those of the Tris egg yolk-based extender (66.67%) and the control of two extenders (53.33% and 46.67%, respectively) as shown in Table 6.

## Discussion

Our study indicated that there were no significant differences in semen quality and functionality between equilibrated controls of two extenders, as well as between equilibrated PRP-supplemented OptiXcell® or Tris egg yolk-based extenders, except for MMP%. This finding is consistent with the results of Chaudhari et al. [39], who also found no significant difference between Tris egg yolk extender and OptiXcell® regarding equilibrated semen quality and functionality, especially during the winter season.

Cryoprotectants in extenders are essential for semen cryopreservation; modifying semen extenders to include biological additions like PRP for sperm preservation may improve sperm qualities and functionality, boost antioxidant activity, and reduce the lipid peroxidation (MDA) profile, ultimately allowing for higher conception rates [10]. This study discovered that spermatozoa cryopreserved in PRP-supplemented OptiXcell® performed significantly better than PRP-supplemented Tris egg yolk-based extenders concerning post-thaw sperm viability, progressive motility, and decrease in abnormalities; this improvement was related to the composition of OptiXcell® and the action of PRP-supplementation, whereas the reversible binding and fusing of OptiXcell® liposomes to spermatozoa during cryopreservation have reduced cryoinjuries to the mitochondria and axonemal machinery [40]. Furthermore, a multitude of bioactive components in PRP, such as FGF, NGF, and TGF, may be connected to this improvement. FGF boosts protein kinase B signaling pathways, extracellular signal-regulated kinase, and the phosphorylation of FGF receptors on sperm flagella, resulting in increased sperm motility proportions [41]. TGF also has anti-inflammatory properties, increases sperm motility, and significantly affects reproduction success [42]. Furthermore, NGF greatly improved sperm viability and motility and reduced necrosis; its reduction resulted in human seminal abnormalities [43]. In contrast, it is hypothesized that decreased sperm viability, motility, and increased sperm abnormality in Tris egg yolk-based extenders compared to OptiXcell® may be caused by a cellular energy production mechanism that is delayed as a result of the presence of high-density lipoproteins, corticosteroid hormones, and other granular substances in egg yolk [44]. Regarding post-thaw motility, the findings of this study are comparable to those of further investigations carried out on buffalo [45] and bovine subjects [46]. The freezing process causes spermatozoa to undergo cryocapacitation because of protein (tyrosine) phosphorylation and calcium ion saturation, eventually destabilizing the sperm plasma membrane [47]. Our findings suggested that the more significant proportion of sperm with an intact acrosomal membrane in PRP-supplemented OptiXcell® compared to PRP-supplemented Tris egg yolk-based extenders may be attributable to the fact that OptiXcell® lessened the effects of cryocapacitation in cryopreserved semen [20]. These findings concur with Stewart et al. [48]and Ansari et al. [40].

Additionally, insulin-like growth factor 1’s actions in PRP, which lessen cryopreservation damage by preserving acrosomal membrane-associated proteins, may be responsible for a higher percentage of sperm with a normal acrosomal membrane [49]. Our results demonstrate that the PRP-supplemented OptiXcell® extender considerably increased the proportion of spermatozoa with an intact plasma membrane following cryopreservation, consistent with Naz et al. [20]. Moreover, as PRP’s protein composition mechanically preserves sperm membranes by decreasing the possibility of ice crystal formation or thawing at different phases of the cryopreservation process, it also adds to the buffering action of PRP, which minimizes osmotic stress [50].

This study found that the post-thaw mitochondrial membrane potential of PRP-supplemented OptiXcell® was much greater than that of PRP-supplemented Tris egg yolk-based extenders. This improvement may be attributable to OptiXcell® having lower sperm abnormalities and cryodamage than Tris egg yolk-based extenders, which are the main factors affecting mitochondrial membrane potential [51]. Additionally, this might be explained by the presence of vascular endothelial growth factor in PRP, which affects mitochondrial potential maintenance and OS protection [52]. This study found that PRP-free OptiXcell® and the PRP-free Tris egg yolk-based extender produced spermatozoa with identical DNA integrity [40]. But in contrast to the control group, PRP supplementation with extenders reduced OS and ROS generation and subsequently improved sperm DNA integrity [53].

In this study, we found that PRP-free Tris egg yolk-based extender had lower antioxidant activity than that of PRP-free OptiXcell® extender; this could be because Tris egg yolks contain fewer antioxidants or because microbial contamination of the egg yolks produced ROS, which could have led to spermatozoa’s lipid peroxidation [54]. PRP affects antioxidant enzymes in mammalian cells to protect cells from damage [55]. Our studies show that the addition of PRP to semen extenders before cryopreservation dramatically raises TAC and GSH levels in buffalo bull semen while reducing MDA levels, proving PRP’s potent antioxidant action by Bader et al. [56], who discovered that 2% PRP could repair the adverse consequences of H_2_O_2_-induced OS on human spermatozoa, and Yan et al. [11], who also found that human sperm that had undergone PRP treatment had lower levels of ROS. The semen quality, functionality, and antioxidant parameters of different concentrations of PRP-supplemented extenders (Tris egg yolk and/or OptiXcell®) increased gradually from 0% PRP (control) to 5% PRP to reach the highest at 10% PRP, then stabilized to decrease at 15%. This may be due to the elevated levels of extender amino acids in PRP components. This leads to increased osmotic pressure and extender hypertonicity, as Ali et al. [57] and Khlifaoui et al. [58] reported. These adverse effects can significantly decrease sperm antioxidant enzyme content, sperm quality, and in vivo fertility.

This study’s in vivo fertilization rate was significantly higher when cryopreserved semen was supplemented with 10% and 15% PRP in the OptiXcell® and Tris egg yolk-based extenders. These results suggest that the supplementation of PRP improves sperm DNA integrity, which in turn can enhance bull sperm fertility. Studies have shown that sperm DNA degradation can hurt fertility [59]. Additionally, the improved fertilization rate may be attributed to the fact that PRP enhances the immune response and has antimicrobial and anti-inflammatory properties, reducing the risk of uterine infection after breeding buffaloes. Uterine infection is a significant cause of poor embryonic survival, and by lowering this risk, PRP can lead to better embryonic recovery and higher pregnancy rates [60].

## Conclusion

Compared to Tris egg yolk-based extender, OptiXcell® was more successful in cryopreserving bull semen. Additionally, extenders supplemented with PRP have properties that defend against cryodamage and improve in vivo fertility. The optimal concentration for cryopreserving buffalo bull semen is a 10% PRP-containing OptiXcell® extender, which enhances post-thaw sperm quality, functionality, antioxidant enzyme activity, and spermatozoa fertility.

## Data Availability

Upon request from the corresponding authors.
